# P01-004 – MEFV genes and FMF

**DOI:** 10.1186/1546-0096-11-S1-A8

**Published:** 2013-11-08

**Authors:** F Salehzadeh, M Jafariasl, S Jahangiri, S Hosseiniasl

**Affiliations:** 1 Pediatric Rheumatology, Bouali Hospital, Iran, Islamic Republic Of; 2 Pediatric Rheumatology, ARUMS, Iran, Islamic Republic Of; 3 Genetic, Emam Khomeini hospital, Ardabil, Iran, Islamic Republic Of

## Introduction

Familial Mediterranean Fever (FMF) is a hereditary autoinflammatory disease with autosomal recessive inheritance pattern often seen in the Turks, Arabs, Armenians and Jews people characterised by recurrent episoded of fever and polyserositis and rash. Recently the definitive diagnosis of FMF determines by MEFV gene analysis.

## Objectives

In this study we analysed twelve MEFV gene mutations inmore than two hundred FMF patients who had Mediterranean fever diagnosis on the basis of clinical Tel – Hashomer criteria.

## Methods

In northwest of IRAN, 216 patients with FMF diagnosis based on Tel-Hashomer criteria, referred to the genetic laboratory to12 common MEFV genes analysis. P369S, F479L, M680I(G/C), M680I(G/A), I692del, M694V, M694I, K695R, V726A, A744S, R761H, E148Q mutations were analysed by using amplification refractory system for 11 of those and the PCR was performed for E148Q.

## Results

Among Of these FMF patients, no mutation was detected in 51(23/62%) patients and 165 (76/38%) patients had one or two mutation. 33 patients (15/28%) homozygous, 86 patients (39/81%) were compound heterozygous, 46 patients (21/29%) were heterozygous. The most common mutation, were M694V (23/61%) V726A (11/11%) and E148Q (9/95%) respectively.

## Conclusion

Common 12 MEFV genes analysis could not detect 50% of our patient who had FMF on the basis of Tel – Hashomer clinical criteria. Therefore it needs more genes analysis in genotyping studies, we conclude that clinical criteria is still the best way in diagnosis of FMF.

## Disclosure of interest

None declared

